# Utilization of AlphaFold2 to Predict MFS Protein Conformations after Selective Mutation

**DOI:** 10.3390/ijms23137235

**Published:** 2022-06-29

**Authors:** Qingjie Xiao, Mengxue Xu, Weiwei Wang, Tingting Wu, Weizhe Zhang, Wenming Qin, Bo Sun

**Affiliations:** 1 Shanghai Advanced Research Institute, Chinese Academy of Sciences, Shanghai 201204, China; xiaoqj@sari.ac.cn (Q.X.); wangww@sari.ac.cn (W.W.); wutt@sari.ac.cn (T.W.); zhangweizhe@sari.ac.cn (W.Z.); 2 Shanghai Institute of Applied Physics, Chinese Academy of Sciences, Shanghai 201800, China; 2018216024@njau.edu.cn; 3 Department of Microbiology, College of Life Sciences, Nanjing Agricultural University, Nanjing 210095, China

**Keywords:** MFS transporter, selective mutation, predicted structure, multiple conformations, high accuracy

## Abstract

The major facilitator superfamily (MFS) is the largest secondary transporter family and is responsible for transporting a broad range of substrates across the biomembrane. These proteins are involved in a series of conformational changes during substrate transport. To decipher the transport mechanism, it is necessary to obtain structures of these different conformations. At present, great progress has been made in predicting protein structure based on coevolutionary information. In this study, AlphaFold2 was used to predict different conformational structures for 69 MFS transporters of *E. coli* after the selective mutation of residues at the interface between the N- and C-terminal domains. The predicted structures for these mutants had small RMSD values when compared to structures obtained using X-ray crystallography, which indicates that AlphaFold2 predicts the structure of MSF transporters with high accuracy. In addition, different conformations of other transporter family proteins have been successfully predicted based on mutation methods. This study provides a structural basis to study the transporting mechanism of the MFS transporters and a method to probe dynamic conformation changes of transporter family proteins when performing their function.

## 1. Introduction

The major facilitator superfamily (MFS) is the largest secondary transporter family and is widely distributed in living organisms [1,2,3]. Studies have shown that members of this family transport a wide range of substrates, including sugars, amino acids, peptides, nucleosides, and drug molecules, across the biomembrane [4,5]. MFS members have similar topology, where the canonical MFS-fold is comprised of 12 transmembrane (TM) helixes, divided into two six-helix bundles with one N-terminal and one C-terminal. These two domains are connected by a long, flexible intracellular loop, and the substrate binding site is located within the cavity formed by the two domains [6,7]. Three major conformations occur during transport of substrates: inward-facing, occluded, and outward-facing [7]. In order to fully understand the transport mechanism, it is necessary to obtain structures of these different conformations. However, it is difficult to acquire multiple conformations as these proteins are generally unstable in detergent-solubilized solution and highly dynamic. This makes it challenging to purify the protein and determine its structure. At present, only a handful of MFS proteins with multiple conformations have been deposited to the Protein Data Bank (PDB) [8,9,10,11,12].

Computational methods for protein structure prediction have made significant progress, due to the increasing number of protein structures in the PDB, the explosion of genome sequencing, and rapid advances in deep learning [13,14,15,16]. In the 14th Critical Assessment of Protein Structure Prediction (CASP14), protein structures predicted by AlphaFold2 can achieve accuracy at the atomic level, based on a new model of neural networks [17]. To date, AlphaFold2 has been used to predict the structure of 350,000 proteins, including proteins expressed in 20 commonly used model organisms, such as *E. coli*, yeast, fruit flies, and even 20,000 human proteins [18]. However, most proteins have only been predicted in a single conformation. This observation stimulated the question of whether membrane protein structures with different conformations can be predicted by multiple point mutations to provide a structural and theoretical basis for studying the transport mechanisms of transporter proteins. In this article, a hypothesis has been investigated by mutating residues at the interaction interface of the N- and C-terminal structural domains of the MSF transporter protein. The results show that the mutagenesis approach is effective in predicting different conformations and is highly consistent with the structure obtained using crystallography. In addition, a script to facilitate the execution of the mutation-based approach to structure prediction has been supplied.

## 2. Methods

### 2.1. Determination of Coevolutionary Residue Pairs of MFS Proteins

MFS protein sequences were obtained from the UniProt database (https://www.uniprot.org/, accessed date: 18 August 2021). The residue–residue contacts in MFS proteins were predicted using GREMLIN (http://gremlin.bakerlab.org/submit.php, accessed date: 1 June 2022) [19,20,21]. Multiple sequence alignment (MSA) was performed using HHblits, where the E-value was set to 1 × 10^−10^ and sequences with less than 70% coverage and Gap values higher than 75% in MSA were filtered out. Coevolutionary residue pair prediction was performed using Vanilla.

### 2.2. Mutation of Amino Acid Residues

The predicted structures were downloaded from the AlphaFold Structure Prediction Database (https://www.alphafold.ebi.ac.uk/, accessed date: 25 June 2022). Pymol 1.4.1 (https://www.pymol.org/, accessed date: 25 June 2022) was used to analyze the mutation site and compute the Cα root-mean-square deviation (RMSD) between structures. ChimeraX was used to prepare the structural Figures [22]. A graphical abstract was prepared with BioRender (created with BioRender.com, accessed date: 23 November 2021). For the outward conformation, the residues were selected for tryptophan mutation on the cytoplasmic side of the interface between the N- and C-terminal domains. For the inward conformation, the residues selected for mutation to tryptophan were located on the periplasmic side of the interface between the N- and C-terminal domains. The mutation sites for the MFS member proteins are detailed in Table S1.

### 2.3. Protein Structure Prediction Using AlphaFold2

AlphaFold2 was downloaded from Github and installed as described (https://github.com/deepmind/alphafold, accessed date: 3 August 2021). Protein structure prediction was performed using the recommended codes [17,18]. Considering that some structures of MFS protein have already been deposited in the Protein Data Bank (PDB), the predicted results may be affected if these structures were used as a template. Therefore, the max template date (before 1 January 2000) was set to avoid using these structures as templates.

### 2.4. Molecular Dynamic Simulation

More than one conformation will be resulted in the prediction once different mutations are applied. All these conformations are supposed to exist in vivo, but verification through structural biology experiments is difficult. To test whether the conformation could be changed naturally, a molecular dynamic simulation was performed to simulate the dynamic process of proteins starting from a selected conformation. The simulation system was built with CHARMM–GUI [23]. The protein (inward conformation, UniProt ID: POAA76) was inserted into POPE membranes with explicit TIP3P water and 0.15 M NaCl (in addition to the counterions used to neutralize charge) by using the CHARMM-GUI web-based graphical interface. Box size was set as 99 × 99 × 112 Å^3^ with 115,590 atoms for the simulation system. GROMACS (2019.3 version) was applied to simulate the system with the CHARMM36 force field. The systems were energy minimized using the steepest descents method over 5000 steps. Then, relaxation was performed by applying restraints in a stepwise manner using the standard CHARMM-GUI equilibration protocol [23]. Production simulations were performed for 100 ns without positional restraints with 2 fs time steps at a temperature of 303 K and constant pressure (1 bar). During the MD process, the LINCS algorithm was used to constrain the bond length [24]. The cutoff distance for non-bonded interactions was set to 12 Å and long-range electrostatic interactions were computed using the particle–mesh Ewald (PME) method [25].

### 2.5. Instruction for Script Usage

We have written a script to implement our method. The script runs as follows: Firstly, the script will generate a user-specified mutated sequence. The original protein sequence and mutation site (sequence number) and type should be input manually. Secondly, the mutated new sequence will be passed to AlphaFold2 to predict the structure. Thirdly, the predicted mutant structure will be reverted to the wild type by using PyRosetta, and the reverted side chain will be optimized [26]. The script will be included in the Supplementary Materials for more information.

## 3. Results

### 3.1. Distribution of Coevolutionary Residues on the Interface of MFS Proteins

**Figure 1:**
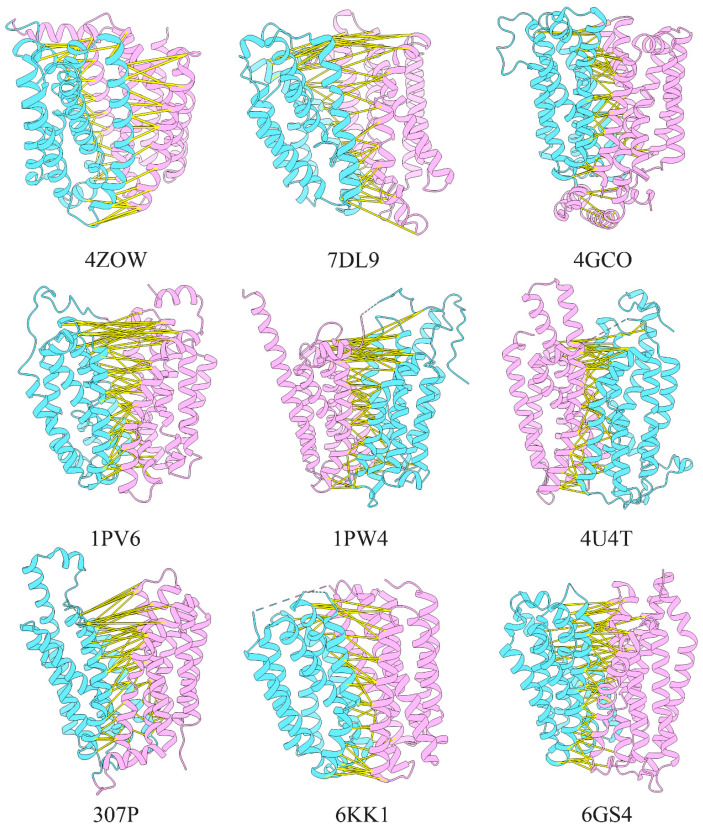
Location of the coevolutionary residue pairs with the Prob score above 0.9 on the interface of N- and C-terminal domains. The yellow stick linked the residue pairs. The N-terminal domain was colored violet, and the C-terminal domain was colored cyan.

Previous studies have demonstrated that close residues in three-dimensional structures tend to have common mutations [21]. These residue pairs can be determined by multiple sequence alignments, and this information can be used to predict protein tertiary structure. In this study, covarying residue pairs for MFS members were predicted by GREMLIN. Here, we focused on MFS proteins expressed in *E. coli* for which three-dimensional structures have been reported. Our results demonstrated that there were many covarying residue pairs distributed on the interface of N- and C-terminal domains with a Prob score (assignment of a probability to every amino acid sequence in the paired alignment [19]) above 0.8, as shown in Figure 1. Several of these covarying residue pairs were distributed on the intracellular and periplasmic sides of the interface between the two domains. Considering the existence of inward and outward conformation in the MFS member, the result indicates the presence of multiple types of conformational information in coevolved residue pairs. This implies that different conformations could be obtained with structure prediction by disturbing these covarying residues on the cytoplasmic or periplasmic sides. Previous studies have also found that mutations can maintain the inward-opening conformation of LacY and XylE by introducing large lateral groups into the periplasmic side of the interface between the N- and C-terminal domains [27,28]. The above analysis supports the development of a method to predict various structural conformations for MFS members by introducing large side-chain residues, such as tryptophan.

### 3.2. Prediction of Mutant Protein Structures

**Figure 2:**
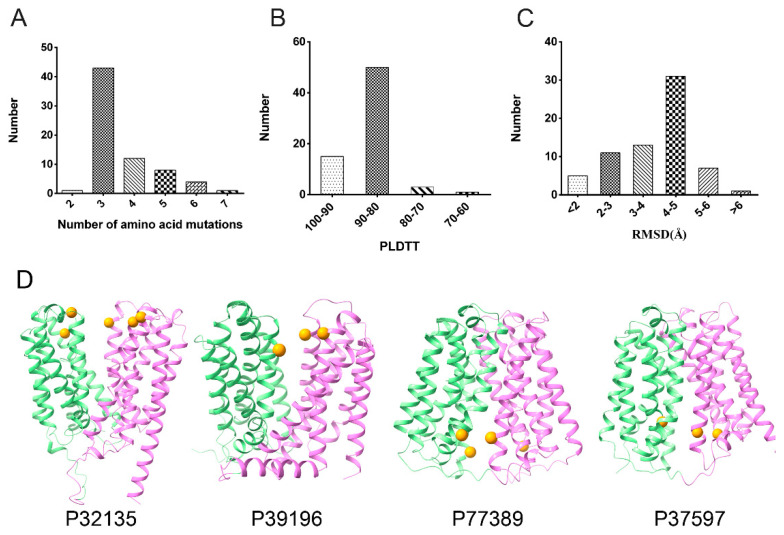
Statistics of 69 MFS members in *E. coli*. (**A**) The number of mutant residues. (**B**) The pLDDT score. (**C**) The Cα RMSD between two conformations. (**D**) The mutant structures were predicted by AlphaFold2. The N-terminal domain was colored pale green, and the C-terminal domain was colored violet. The mutation sites were represented by orange spheres.

The conformational changes in the MFS of transporters often involve alternating openings of the N- and C-terminal structural domains, with many coevolutionary residues distributed over residues at the N- and C-terminal interaction interfaces. Mutagenesis is applied to alter the multiple sequence alignment information (MSA), which in turn affects the information of the original coevolving residue pairs, thus predicting a different conformation. Residues with smaller side chains on the intracellular or periplasmic surfaces were mutated to tryptophan. AlphaFold2 was then utilized to predict conformational changes in *E. coli* MFS proteins. At the beginning stage of our trial, only 40 proteins had obvious conformational changes when only three residues were mutated. We then redesigned or increased the number of mutation sites for those proteins without obvious conformational changes by introducing the coevolutionary data of residue pairs. Finally, our results demonstrated that 69 out of 74 total MFS proteins had an obvious change in conformation compared to the wild type after several rounds of residue mutation. The mutation sites for these proteins are listed in Table S1. Here, one protein changed conformation after two sites were mutated, 43 proteins after three mutations, 12 after four mutations, and 13 proteins required greater than four residues mutated to tryptophan (Figure 2A). The quality of the predicted models was assessed using the mean value of their pLDDT (predicted Local Distance Difference Test) score. The pLDDT is a per-residue measure that estimates the consistency between the predicted and experimental structure based on the local distance difference in Cα [29]. Previous studies have shown that a pLDDT value above 70 generally corresponds to an accurate backbone prediction [18]. Fifteen of our predicted protein models scored between 90 and 100 while 50 scored between 80 and 90. Of the remaining four protein models, only one scored below 70, as shown in Figure 2B. The above results indicate that the most predicted structures have a high degree of confidence.

Most of the structures showed obvious conformational changes as noted by RMSD above 2 Å when superimposing the two conformations (Figure 2C). However, a smaller RMSD (<1) was exhibited when the N- and C-terminal domains were superimposed, respectively, suggesting that the amino acid mutation had no obvious change in the N- and C-terminal domains. In order to intuitively observe these mutation sites, Figure 2D depicts a few of the predicted structures with the location of the mutation sites represented by spheres.

### 3.3. Comparison of the Structures from Prediction and Experiment

**Figure 3:**
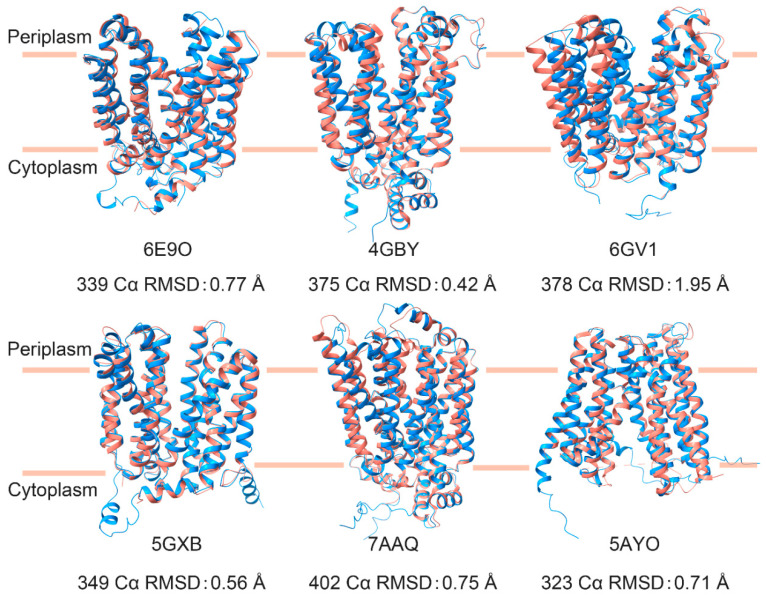
Structural alignment between the predicted structure (mutant, dodger blue) and the experimental structure (salmon). The PDB numbers corresponding to the UniProt ID are 6E9O and P0AA76, 4GBY and P0AGF4, 6GV1 and P0AEY8, 5GXB and P02920, 7AAQ and Q9LT15, and 5AYO and Q6MLJ0.

To date, several structures of MFS proteins (UniProt ID: P0AA76, P0AEY8, P0AGF4, P02920, Q6MLJ0, and Q9LT15) have been found in both the outward- and inward-opening conformations [9,10,11,12,30,31,32,33,34,35,36]. There is an obvious difference when superimposing the two conformations, denoted with an RMSD above 3 Å. Furthermore, our predicted structures were superimposed with the experimentally determined structure. The RMSD between the predicted structure and the experimental structure was 0.84 Å (inward conformation, PDB ID: 6E9N) and 0.77 Å (outward conformation, PDB ID: 6E9O) for P0AA76, 1.01 Å (inward, PDB ID: 4QIQ) and 0.42 Å (outward, PDB ID: 4GBY) for P0AGF4, 1.12 Å (inward, PDB ID: 1PV6) and 0.56 Å (outward, PDB ID: 5GXB) for P02920, 0.71 Å (inward, PDB ID: 5AYO) and 0.83 Å (outward, PDB ID: 5AYM) for Q6MLJ0, 0.88 Å (inward, PDB ID: 7AAR) and 0.75 Å (outward, PDB ID: 7AAQ) for Q9LT15, and 0.39 Å (inward, PDB ID: 4ZP0) and 1.95 Å (outward, PDB ID: 6GV1) for P0AEY8. Most of these predicted structures, including mutants, have approximately 1 Å or less deviation from the experimentally determined structures in the PDB. Figure 3 shows the superimposition between the predicted structures of our mutations and the experimental structure. Of these structures, only P0AEY8 had an RMSD greater than one angstrom (1.95 Å) when superimposing the predicted outward structure with the experimental equivalent. This difference is attributed to the different degrees of outward openings between the two structures. The RMSD of the N- and C-terminal domains for the predicted structure and experimentally determined structure was less than 0.5 Å. It is worth mentioning that the structure of Q9LT15 was not used as a training set of AlphaFold2 as the structure was reported in August 2021, after the release of AlphaFold2. The above analysis shows the high accuracy in structure prediction with AlphaFold2 for the different conformations induced by our mutations.

### 3.4. Prediction of Other Family Transporters

**Figure 4:**
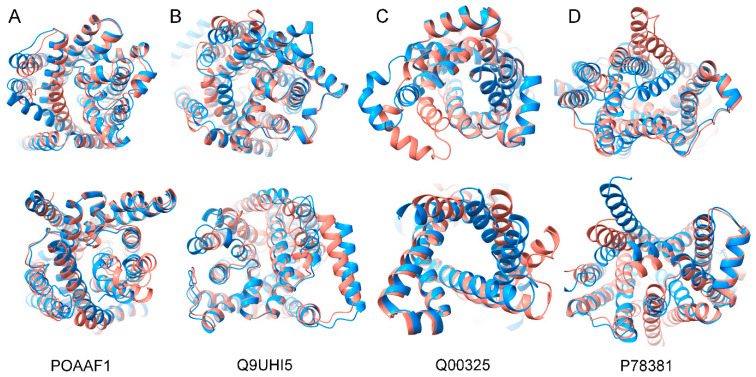
Structural alignment of the other transporters between the predicted structure of the wild type (salmon) and mutant (dodger blue) with the UniProt numbers POAAF1 (**A**), Q9UHI5 (**B**), Q00325 (**C**), and P78381 (**D**).

In addition, we used mutation methods to predict different conformations of other family transporters including members of the amino acid-polyamine-organocation (APC) superfamily (UniProt ID: P0AAF1, Q9UHI5), mitochondrial carrier family (UniProt ID: Q00325), and nucleotide-sugar transporter family (UniProt ID: P78381). Protein members of these families have significantly different topologies and do not have obvious N- and C-terminal domains compared to the MFS proteins, but there are inward- and outward- opening conformations during substrate transport. After selectively mutating these residues with smaller side chains on the closed side from the wild-type structure, AlphaFold2 was then used to predict conformational changes. The results showed that these mutants had a conformational change compared to the wild type, that is, outward to inward conformation (UniProt ID: P0AAF1, Q00325) and inward to outward conformation (UniProt ID: Q9UHI5, P78381) (Figure 4). The RMSD of Ca between the wild-type structure and mutant structure was 1.63 Å (UniProt ID: P0AAF1), 2.17 Å (UniProt ID: Q9UHI5), 2.77 Å (UniProt ID: Q00325) and 3.0 Å (UniProt ID: P78381), respectively, by superposing the outward and inward conformations. It is worth mentioning that the two proteins (UniProt ID: Q9UHI5 and P78381) did not obtain the conformation of the mutant with the experimentally determined structure, but it was found for the structure of other members of the same family with similar conformations with the mutant. The structure of the mutant (UniProt ID: Q9UHI5) was in the same conformation as the member (PDB ID: 6I1R) of the same family, with only an RMSD of about 1.11 Å (Figure S1). The protein of the mutant (UniProt ID: P78381) was in the same conformation as the member (PDB ID: 7DSK) of the same family, with only an RMSD of about 1.04 Å (Figure S1). According to the above analysis, AlphaFold2 can be used to predict different conformations for other family transporters after selective mutation.

## 4. Discussion

The AlphaFold2 algorithm has been reported to predict the three-dimensional conformation of target proteins by obtaining amino acid coevolutionary information through multiple sequence alignment (MSA) [17]. Consequently, coevolutionary information among interfering residues provides an idea to obtain multiple conformations of target proteins. Excitingly, the feasibility of this approach was confirmed by the recent work of Richard A. Stein et al. [37], who obtained information on different coevolving residue pairs by varying the depth of MSA to predict the conformation of multiple family proteins. Their results also further demonstrate the accuracy of AlphaFold2 in predicting protein structures. Coincidentally, Alamo et al. recently predicted different conformations of some transporters through AlphaFold2, which provided a basis for our research [38]. Differently, in our study, we focused on the interference of MSA information by mutating multiple residues, thus affecting coevolving residue pairs.

For the targeted mutation of residues, we mainly selected residues at the interface of the N- and C-terminal structural domain interaction. We introduced spatial blocking into the original conformation by mutating the small-side-chain residues to large-side-chain residues to increase the probability of obtaining a different conformation. In addition, we modified some of the MFS members by mutating corresponding residue to alanine (compared with mutation to tryptophan). We found that some of the predicted structures had no conformational change (P0AAF, P28246, and P76198) or only one of the five structures given by AF2 had obvious conformational change (Q47142, P32135, and P77549), which indicates a low success rate of obtaining target structures with significant conformational changes. These results suggest that mutating to tryptophan could increase the probability of obtaining a different conformation. In our mutation experiments, we also analyzed the minimum number of mutation sites required to successfully obtain a different conformation and found that in most cases only three residue sites were sufficient. Overall, the success rate of obtaining different conformations with selective mutagenesis is above 90%, indicating that the mutation-based approach is very effective in obtaining different conformations of MFS members. In addition, we have successfully applied this method to other transporter family members, showing that our approach is not limited to MFS proteins.

Multiple conformations of MFS proteins provide an intuitive understanding of their dynamics of substrate transport [7]. Our studies have shown that the mutation of residues on one side of the protein to tryptophan can orient the two domains closer to each other on the other side, although this does not induce a large conformational change. This provides the intermediate state conformation during substrate transport. Multiple conformations have been obtained for several MFS member proteins, including P0AA76, P37662, and P60778. The transition from the inward-opening to the outward-opening conformation was illustrated by superposing the C-terminal domain of these structures (Figure S2). Comparison of the two P0AA76 inward-facing conformations showed that selective mutations of residues on the extracellular side resulted in only minor changes between the two domains on this side, but significant conformation changes occurred on the intracellular side (Figure S2). This might suggest that small local rearrangements might be the switch for conformational change. Compared with the other two proteins, P0AA76 had a more significant intermediate conformation. Then, the 100 ns molecular dynamics were conducted for the protein with inward conformation to test whether it could change to another conformation automatically. The MD results visually showed the transiting process of conformation from inward to occluded conformation during 100 ns (Figure S3A). The occluded conformation was similar to the intermediate state that was predicted with Alphafold2. Snapshots of 65 ns and 70 ns were superposed with the predicted conformation that showed RMSD of Cα below 1 Å (Figure S3B). Based on the above analysis, it is believed that Alphafold2 can be used to explore the potential conformation of proteins by applying selective mutations.

Although some progress has been made, there are still some limitations in our research. First, although our method has shown some advantages in MFS and a few other transporter families, further validation of the applicability is needed because of the large variety of transporter families. Second, the number of transporter structures currently stored in the PDB database is relatively small, especially for the proteins with multiple conformations. Thus, predicted structures provided by our method need to be validated and supported by experimental methods to further investigate their functions. Finally, the selection of mutation sites in this study was based on MSA. However, it remains a challenge whether the location and number of these mutation sites are sufficient to help obtain the different conformations. In this study, we found that some proteins (P32135, P37662, and P60778) required multiple rounds of mutation site design to obtain different conformations. Therefore, it remains to be investigated how to effectively extract different conformations from MSA and develop better algorithms for different conformational structure prediction.

## 5. Conclusions

Transporters play an important role in cell growth, metabolism, intracellular environmental homeostasis, and signal transduction [39,40]. Furthermore, these proteins are also closely associated with drug resistance and disease development in many pathogenic bacteria, making the study of their transport mechanisms important for the treatment of many diseases and the development of drugs [41,42]. Currently, studies of the transport mechanisms of members of the transporter families are mainly based on structural biology, that is, by obtaining multiple conformational structures of target proteins to elucidate their transport mechanisms.

Here, we provide a method for obtaining the different conformations of transporters. Our method is based on AlphaFold2 prediction and the mutation at key sites of the target protein. Currently, we have successfully predicted multiple conformations of MFS members in *E. coli* with this method, and it is demonstrated that the method is applicable to other families of transporters as well. Inward and outward are the main conformations of the transporters, which are the main models for the structure-based drug discovery. In our research, the inward and outward conformations of MFS transporters were successfully predicted through the AlphaFold2 algorithm. Hence, our research is important for exploring the transport mechanisms of transporters as well as structure-based drug design and screening. Although our current study still has some limitations, it provides a direction for the development of fully automated prediction of different conformations.

## Data Availability

Data available on request from the authors. The data that support the findings of this study are available from the corresponding author [B.S.], upon reasonable request.

## Supplementary Materials

The following supporting information can be downloaded at: https://www.mdpi.com/article/10.3390/ijms23137235/s1.
