# Supplementary material for: Utilization of AlphaFold2 to Predict MFS Protein Conformations after Selective Mutation
Source: Int J Mol Sci. 2022 Jun 29;23(13):7235. doi: 10.3390/ijms23137235 (PMC9266783; doi:10.3390/ijms23137235)
Supplement: Supplementary file 1 [file ijms-23-07235-s001.zip › ijms-1767301-supplementary.pdf]

Supplementary Information

Utilization of AlphaFold2 to Predict MFS Protein Conformations after Selective Mutation

Qingjie Xiao [1,†], Mengxue Xu [2,3,†], Weiwei Wang [1], Tingting Wu [1], Weizhe Zhang [1], Wenming Qin [1,*] and Bo Sun [1,2,*]

[1] Shanghai Advanced Research Institute, Chinese Academy of Sciences, Shanghai 201204, China; xiaoqj@sari.ac.cn (Q.X.); wangww@sari.ac.cn (W.W.); wutt@sari.ac.cn (T.W.); zhangweizhe@sari.ac.cn (W.Z.)

[2] Shanghai Institute of Applied Physics, Chinese Academy of Sciences, Shanghai 201800, China; 2018216024@njau.edu.cn

[3] Department of Microbiology, College of Life Sciences, Nanjing Agricultural University, Nanjing 210095, China

* Correspondence: qinwenming@sari.ac.cn (W.Q.); sunb@sari.ac.cn (B.S.)

† These authors contributed equally to this work.

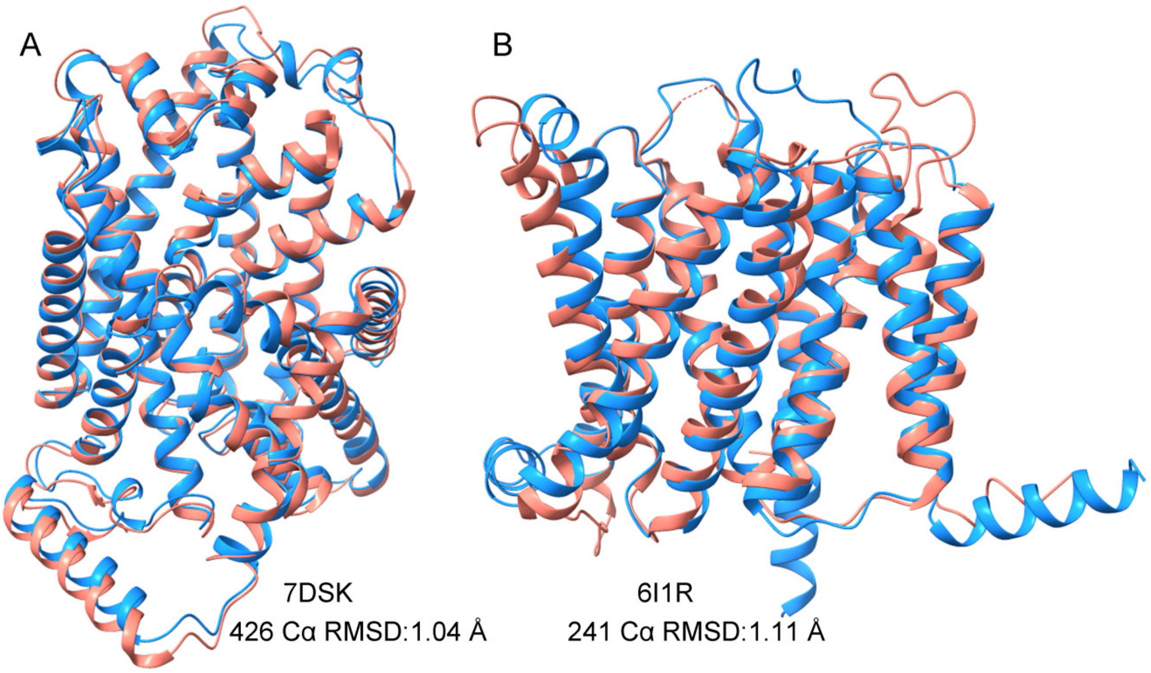

Figure S1. The compare for the predicted structure and the experimental structure. (A) The superposition for the structure of mutant (UniProt ID: P78381) and the member (PDB ID: 7DSK) of the same family. (B) The superposition for the structure of mutant (UniProt ID: Q9UHI5) and the member (PDB ID: 6I1R) of the same family. The dodger blue cartoon showed the mutant, and The salmon cartoon showed the experimental structure.

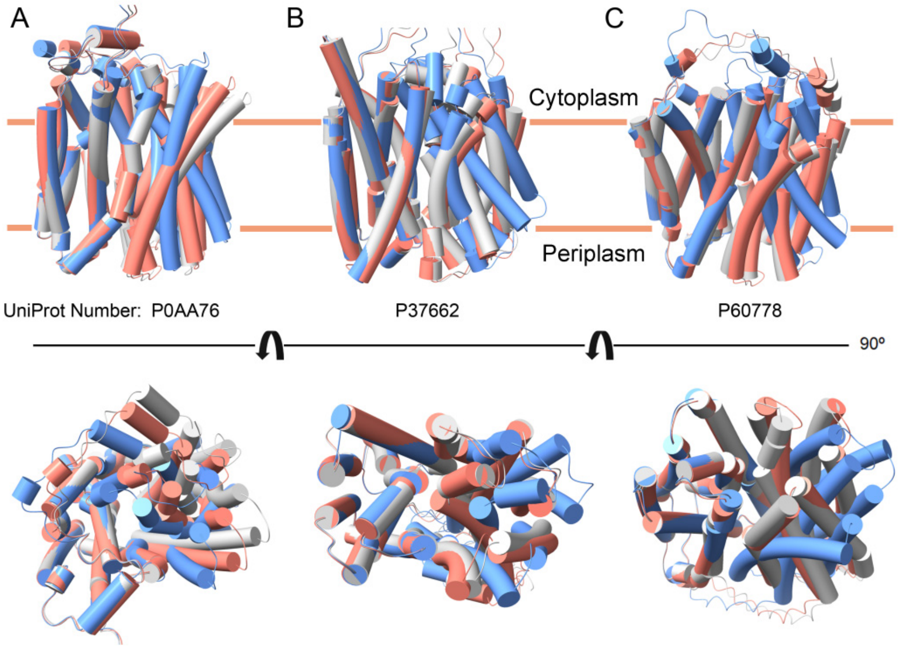

Figure S2. Superposition of multiply conformations. The Uniprot ID are P0AA76 for (A), P37662 for (B), P60778 for (C). The dodger blue cartoon showed the mutant with the A36/S39/ I161/P264/N265 for P0AA76, S31/S46/G235/V256/S357 for P37662, and G28/S43/S46/A153/P230 for P60778. The salmon cartoon showed the mutant with the S64/G134/D275 for P0AA76, S39/I161/N265 for P37662, and V42/P149/230 for P60778. the silver cartoon showed wild type.

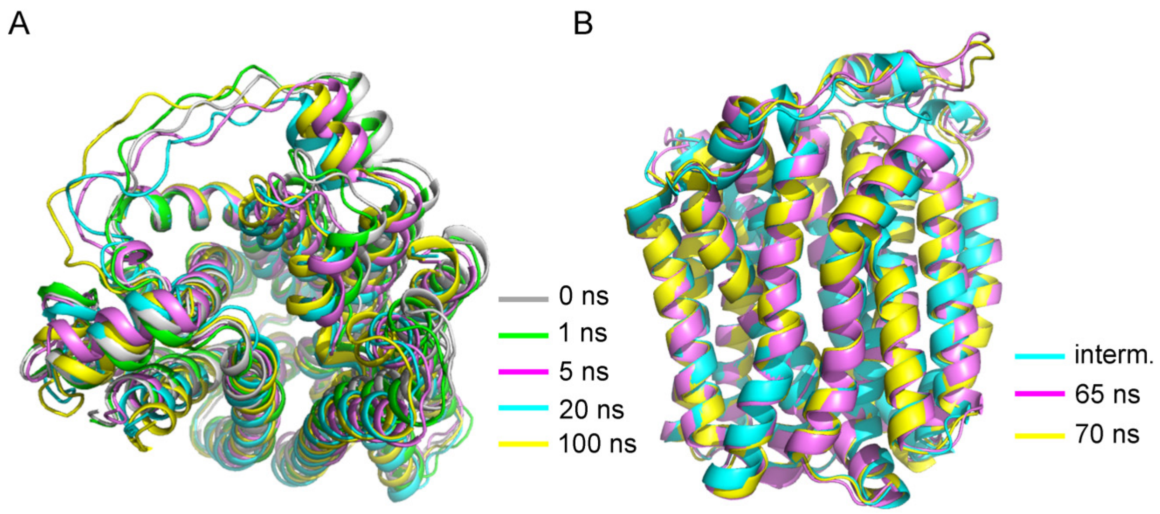

Figure S3. MD for P0AA76 with the inward conformaton. (A) Superposition of structure from 0 ns, 1 ns, 5 ns, 20 ns, 100 ns. (B) Superposition of structure for the predicted intermediate conformation and MD structure. Interm refer to the intermediate conformation.

Table S1. Statistics for mutation site, RMSD and pLDDT score of 69 MFS members in E. coli and 2 MFS members in other species.

Number	Mutation site	Conformation (wild type)	Conformation (mutant)	PLDTT	RMSD (Å)
P39196	P52/V240/T256	inward	outward	94.0	4.05 (383)
P23910	L26/T148/P224	inward	outward	92.9	3.90 (378)
Q47142	G347/S28/S144	inward	outward	92.1	4.97 (379)
P77389	G112/A128/D262/S328	outward	inward	92.10	4.21 (384)
P32135	I32/L154/P243/G257/D376	inward	outward	91.6	5.74 (407)
P28246	P31/L33/P237	inward	outward	91.5	3.84 (372)
P37758	S54/P296/P419	inward	outward	91.5	3.58 (454)
P39386	P34/P51/V235	inward	outward	91.1	3.82 (379)
P0AFF4	S27/ T233/S254	inward	outward	91.0	4.79 (415)
P0AEY8	P38/P55/A240/P243	inward	outward	90.5	2.59 (358)
P10903	V60/T78/P422	Inward occluded	outward occluded	90.5	1.40 (452)
P0ADL1	A130/P143/T331	outward	inward	90.4	5.20 (388)
P69367	V38/A45/P232	inward	outward	90.4	4.60 (391)
P37662	S31/S46/G235/V256/S357	occluded	outward	90.3	2.60 (382)
P76628	G49A235/G251/A357	inward	outward	90.1	4.36 (376)
P37597	V113/A319/A333	outward	inward	89.9	5.08 (387)
P52067	P132/S135/S151	outward	Inward	89.9	3.85 (402)
Q46916	S40/A167/G405	Inward	outward	89.8	5.45 (450)
P31122	S37/T47/L251	inward	outward	89.3	3.37(384)
P39352	A122/V129/P328	outward	inward occluded	89.3	2.84 (398)
P0AE24	S138/I382/S385	occluded	Inward	89.2	1.85 (421)
P0AA76	A36/S39/ I161/ P264/N265/	inward	outward	89.1	4.38 (428)
P76198	P153/P237/P356	inward	outward	89	5.93 (419)
P37621	G56/A167/T239/A254	inward	outward	89	4.60 (389)
P60778	G28/S43/S46/A153/P230	inward	outward	89	4.67 (393)
P21503	T27/A34/L225	inward	outward	88.9	4.96 (370)

P77549	T135/S146/P321	outward	Inward	88.6	4.05 (386)
Q46909	A56/P271/P391	inward	outward	88.5	3.76 (420)
P77726	G337/A122/S325	outward	inward	88.5	4.43 (415)
P0AA80	S38/ S165/I275	inward	outward	88.4	5.67 (438)
P0AEP1	L40/E55/T162/T293	inward	outward	88.3	2.36 (424)
P0AGC0	A176/T283/V284	inward	outward	88.2	4.01(440)
P25744	P125/P330/S350	outward	Inward	88.2	4.36 (393)
P0AGF4	V35/A62/P301/T318	Inward	outward	88.1	3.70 (456)
P45562	S27/P233/S254	inward	outward	87.9	4.38 (409)
P31126	A119/T131/A333	outward	inward	87.6	4.89(394)
P31474	L125/L380/S389	outward	inward	87.5	3.66 (430)
P76269	S123/T126/S379	outward	inward	87.4	3.71 (433)
P02920	P31/T258/G370	inward	outward	87.3	4.27 (407)
P0AEJ0	I55/D163/V289/Q296	inward	outward	87.3	2.30 (406)
P36554	L121/T137/S388	outward	inward	87.2	3.92 (433)
P17583	A38/P220	inward	outward	87.1	3.14 (384)
P76230	A47/P288/P406	inward	outward occluded	87.0	2.46 (386)
P36837	G35/V39/P164/V312/A435	Outward	occluded	86.9	1.45 (469)
P0AA78	T44/P151/ L252	inward	outward	86.8	4.37 (414)
P38055	A133/A149/P376	outward	inward	86.7	5.07(445)
P43531	P57/P60/D185/V277	inward	outward	86.4	4.17 (407)
P76242	P35/T55/P235	inward	outward	86.2	4.51 (384)
P52600	P42/P296/V293/	inward	outward	86.1	2.03 (400)
P77589	G138/A338/S322	outward	inward	86.1	4.74 (395)
P0C0L7	L69/P283/V298	occluded	outward occlude	85.8	1.16 (390)
P76470	I126/A139/P296 /I318	outward	inward	85.7	4.33 (426)
P76350	A46/L274/P397	occluded	outward	85.5	2.93 (425)
P76417	T245/G40/V23/A47/T249	outward	inward	85.5	4.30 (419)
P37643	D313/D89/L148/A368	outward	outward occluded	85.4	1.50 (386)
P41036	A130/S146/P183	outward	occluded	85.0	0.975 (413)

P31436	S38/P241/T255	inward	outward	84.6	4.14 (386)
P76197	P118/T322/T336	outward	Inward	84.5	4.27 (394)
P0AEX3	A146/T147/G159/A162/D304/A362/A371	outward	inward	84.36	2.69 (388)
P0AE16	I126/T133/A339/A351/L340	outward	outward	84.2	6.30 (477)
P75742	D44/P155/G288/A311	inward	outward	84.02	4.62 (478)
P31675	I236/S37/P339	inward	outward	83.7	4.436(385)
P31462	D63/A126/S130/S314/G318/S328	outward	occluded	83.5	3.07 (377)
P77304	G42/E156/S59/P171/P296/T297	inward	outward	82.5	2.92 (477)
P11551	T143/T316/S384/S370	outward	inward	81.7	4.13(407)
P33026	I40/P50/L241	inward	outward	78.4	4.47 (388)
P09836	S89/P137/A140/T144/A370/T382	outward	occlude	73.9	2.56 (426)
P08194	P53/E282/S292/D409/P173	inward	outward	71.5	4.03 (425)
P31442	P29/Q46/P149/G231/S242/A355	inward	outward	66.8	4.76 (382)
P31679	Failed				
P39398	Failed				
P39276	Failed				
P77228	Failed				
P24077	Failed				
Q6MLJ0 (no *E.coli*)	P143/L151/G372	outward	inward	91.53	6.20 (439)
Q9LT15 (no *E.coli*)	T88/P308	inward	outward	76.07	2.12 (450)
P0AAF1	A88/A211/T295	outward	inward	91.46	1.6 (373)
Q9UHI5	Q240/I388/G392	inward	outward	84.45	2.17 (433)
Q00325	G69/E142/T231	outward	inward	69.5	2.77（321）
P78381	A67/I133/A138	inward	outward	79.23	3.00 (294)

*The first column is the Uniprot number, the second column is the residue mutated to tryptophan, the third column and fourth are the wild-type conformation and mutant conformation predicted by AlphaFold2 respectively, and the fifth column is the RMSD for Cα atoms of main chain between the two conformations.
